# Supplementary material for: The Combined Anti-Aging Effect of Hydrolyzed Collagen Oligopeptides and Exosomes Derived from Human Umbilical Cord Mesenchymal Stem Cells on Human Skin Fibroblasts
Source: Molecules. 2024 Mar 26;29(7):1468. doi: 10.3390/molecules29071468 (PMC11013016; doi:10.3390/molecules29071468)
Supplement: Supplementary file 1 [file molecules-29-01468-s001.zip › molecules-2875894-supplementary.pdf]

Supplementary Information: The Combined Anti-aging Effect of Hydrolyzed Collagen Oligopeptides and Exosomes Derived from Human Umbilical Cord Mesenchymal Stem Cells on Human Skin Fibroblasts

Table S1. List of primers for real-time RT-PCR.

**Gene**	**Primer**	**Sequence (50 –30)**
GAPDH	Sense	CAGGAGGCATTGCTGATGAT
	Antisense	GAAGGCTGGGGCTCATTT
p16	Sense	GATCCAGGTGGGTAGAAGGTC
	Antisense	CCCCTGCAAACTTCGTCCT
p21	Sense	TGTCCGTCAGAACCCATGC
	Antisense	AAAGTCGAAGTTCCATCGCTC
p53	Sense	CCTCCTCAGCATCTTATCCGAGTG
	Antisense	CCAACCTCAGGCGGCTCATAG
Lamin B1	Sense	AGGCGAAGAAGAGAGGTTGAAGC
	Antisense	GTGGCTGAGGCGGAATGAGAG

Table S2. Amino acid composition of HCOPs from salmon skin

Amino Acid	Result
Aspartic Acid (Asp)	7.692
Threonine (Thr)	3.299
Serine (Ser)	5.688
Glutamic Acid (Glu)	11.311
Glycine (Gly)	18.949 (20.6%)
L-Alanine (Ala)	13.205 (14.4%)
Valine (Val)	2.947
Cystine(Cys)2	0.215
Methionine (Met)	1.943
Isoleucine (Ile)	1.740
Leucine (Leu)	3.736
Tyrosine (Tyr)	0.294
Phenylalanine (Phe)	0.761
Histidine (His)	1.247
Lysine (Lys)	4.685
L-Arginine (Arg)	8.121 (8.8%)
Proline (Pro)	6.179 (6.7%)
Total amino acid	92.012
Hydrophobic amino acids	49.46 (53.8%)

Table S3. Effect of HucMSC-Exos, HCOPs, and HucMSC-Exos + HCOPs on ECM

construction-related proteins and SASP in HSFs.

		Control	HucMSC-Exos	HCOPs	HucMSC-Exos + HCOPs
Collagen I	Concentration (ng/mL)	2.78±0.23c	3.37±0.13b	3.48±0.06b	4.19±0.39a
	Percentage of control %	100.0±8.3c	121.3±4.7b	125.4±2.2b	151.1±14.1a
Collagen III	Concentration (ng/mL)	216.8±13.1c	280.9±3.2b	284.1±7.2b	316.9±1.5a
	Percentage of control %	100.0±6.0c	129.6±1.5b	131.0±3.3b	146.1±0.7a
MMP-1	Concentration (ng/mL)	7.95±0.19a	4.75±0.09c	5.59±0.11b	3.45±0.15d
	Percentage of control %	100.0±2.5a	59.7±1.2c	70.3±1.4b	43.4±1.9d
MMP-3	Concentration (pg/mL)	12654.5±324.5a	10097.4±178.0c	11515.8±102.7b	8203.7±158.1d
	Percentage of control %	100.0±2.6a	79.8±1.4c	91.0±0.8b	64.8±1.3d
MMP-9	Concentration (ng/mL)	119.0±4.9a	61.3±1.3c	73.9±1.8b	41.9±2.6d
	Percentage of control %	100.0±4.2a	51.5±1.1c	62.1±1.5b	35.2±2.2d
TNF-α	Concentration (pg/mL)	1268.9±16.3a	726.8±17.8c	849.5±28.1b	602.1±70.6d
	Percentage of control %	100.0±1.3a	57.3±1.4c	66.9±2.2b	47.5±5.6d
IL-1β	Concentration (pg/mL)	90.4±2.0a	78.0±1.4c	84.4±0.6b	68.0±1.9d
	Percentage of control %	100.0±2.2a	86.3±1.5c	93.3±0.7b	75.1±2.1d

The data shown are expressed as the mean ± SD from three replicates. Different lowercase letters on bars indicate significant differences among samples ($p < 0.05$).

Table S4. Effect of HucMSC-Exos, HCOPs, and HucMSC-Exos + HCOPs on the expression of p16, p21, p53, and lamin B1 genes in HSFs.

		Control	HucMSC-Exos	HCOPs	HucMSC-Exos + HCOPs
p16	Relative mRNA expression level	1.00±0.02a	0.67±0.08b	0.62±0.00b	0.48±0.05c
	Percentage of control %	100.0±2.2a	66.9±7.6b	62.3±0.6b	47.5±5.2c
p21	Relative mRNA expression level	1.00±0.02a	0.68±0.05b	0.75±0.07b	0.48±0.12c
	Percentage of control %	100.0±1.8a	67.9±5.1b	75.1±7.2b	48.1±11.7c
p53	Relative mRNA expression level	1.00±0.09a	1.05±0.06a	0.83±0.05b	0.72±0.03cb

	Percentage of control %	100.0±9.4a	105.3±6.0a	83.1±4.6b	71.6±3.0cb
LaminB1	Relative mRNA expression level	1.00±0.09c	1.24±0.07b	0.94±0.14c	1.76±0.06a
	Percentage of control %	100.0±9.3c	124.4±7.1b	93.9±14.4c	176.3±6.3a

The data shown are expressed as the mean ± SD from three replicates. Different lowercase letters on bars indicate significant differences among samples ($p < 0.05$).

Table S5. Effect of HucMSC-Exos, HCOPs, and HucMSC-Exos + HCOPs on the expression of p16, p21, and p53 proteins in HSFs.

		Control	HucMSC-Exos	HCOPs	HucMSC-Exos + HCOPs
p16	Relative expression	1.00±0.08a	0.79±0.03b	0.67±0.07b	0.36±0.14c
	Percentage of control %	100.0±7.9a	79.0±3.3b	67.0±6.8b	35.9±14.3c
p21	Relative expression	1.00±0.03a	0.71±0.06b	0.70±0.11b	0.39±0.01c
	Percentage of control %	100.0±3.0a	70.6±5.5b	70.3±11.4b	39.5±1.4c
p53	Relative expression	1.00±0.07a	0.83±0.10b	0.75±0.04b	0.43±0.05c
	Percentage of control %	100.0±7.4a	82.8±9.8b	74.5±4.2b	43.2±4.5c

The data shown are expressed as the mean ± SD from three replicates. Different lowercase letters on bars indicate significant differences among samples ($p < 0.05$).

Figure S1. Effect of passage number on cell-cycle distribution of HSFs

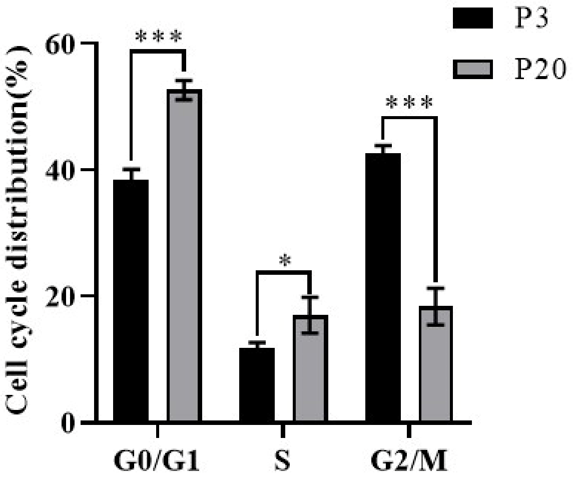

The data shown are expressed as the mean ± SD from three replicates. * $p < 0.05$; *** $p < 0.001$.
